# Effect of Oversulfation on the Composition, Structure, and In Vitro Anti-Lung Cancer Activity of Fucoidans Extracted from *Sargassum aquifolium*

**DOI:** 10.3390/md19040215

**Published:** 2021-04-12

**Authors:** Hui-Hua Hsiao, Tien-Chiu Wu, Yung-Hsiang Tsai, Chia-Hung Kuo, Ren-Han Huang, Yong-Han Hong, Chun-Yung Huang

**Affiliations:** 1 Faculty of Medicine, Kaohsiung Medical University, Kaohsiung 80708, Taiwan; huhuhs@kmu.edu.tw; 2 Center for Cancer Research, Kaohsiung Medical University, Kaohsiung 80708, Taiwan; 3 Center for Liquid Biopsy and Cohort Research, Kaohsiung Medical University, Kaohsiung 80708, Taiwan; 4 Division of Hematology and Oncology, Department of Internal Medicine, Kaohsiung Medical University Hospital, Kaohsiung Medical University, Kaohsiung 80756, Taiwan; 960552@ms.kmuh.org.tw; 5 Cancer Center, Kaohsiung Medical University Hospital, Kaohsiung Medical University, Kaohsiung 80756, Taiwan; 6 Department of Seafood Science, National Kaohsiung University of Science and Technology, No. 142, Haijhuan Rd., Nanzih District, Kaohsiung City 81157, Taiwan; yht@nkust.edu.tw (Y.-H.T.); kuoch@nkust.edu.tw (C.-H.K.); 7 Mackay Memorial Hospital Emergency Department, No. 92, Sec. 2, Zhongshan North Rd., Taipei City 10449, Taiwan; lisa68850@gmail.com; 8 Department of Nutrition, Yanchao Campus, I-Shou University, No. 8, Yida Rd., Jiaosu Village, Yanchao District, Kaohsiung City 82445, Taiwan

**Keywords:** anti-lung cancer, apoptosis, brown algae, fucoidan, human lung carcinoma A-549 cells, oversulfation, *Sargassum aquifolium*

## Abstract

Intensive efforts have been undertaken in the fields of prevention, diagnosis, and therapy of lung cancer. Fucoidans exhibit a wide range of biological activities, which are dependent on the degree of sulfation, sulfation pattern, glycosidic branches, and molecular weight of fucoidan. The determination of oversulfation of fucoidan and its effect on anti-lung cancer activity and related signaling cascades is challenging. In this investigation, we used a previously developed fucoidan (SCA), which served as a native fucoidan, to generate two oversulfated fucoidan derivatives (SCA-S1 and SCA-S2). SCA, SCA-S1, and SCA-S2 showed differences in compositions and had the characteristic structural features of fucoidan by Fourier transform infrared (FTIR) and nuclear magnetic resonance (NMR) analyses. The anticancer properties of SCA, SCA-S1, and SCA-S2 against human lung carcinoma A-549 cells were analyzed in terms of cytotoxicity, cell cycle, Bcl-2 expression, mitochondrial membrane potential (MMP), expression of caspase-3, cytochrome *c* release, Annexin V/propidium iodide (PI) staining, DNA fragmentation, and the underlying signaling cascades. Our findings indicate that the oversulfation of fucoidan promotes apoptosis of lung cancer cells and the mechanism may involve the Akt/mTOR/S6 pathway. Further in vivo research is needed to establish the precise mechanism whereby oversulfated fucoidan mitigates the progression of lung cancer.

## 1. Introduction

Lung cancer is the most common cancer worldwide and has high morbidity and mortality rates. Thus, considerable research efforts have been undertaken aimed at improving the prevention, diagnosis, and treatment of this disease [1]. The biggest risk factors for lung cancer are habitual smoking of tobacco, air pollution (indoor and outdoor), radiation, and occupational exposure to hazardous chemicals [2]. Lung cancer is the most prevalent form of cancer in Taiwan and is the most common cause of cancer-related mortality [3]. While therapeutic approaches for lung cancer have seen significant advances in recent years, the treatment of this disease remains a considerable clinical challenge. Hence, in order to improve patient outcomes, there is a crucial need for novel agents and targets for the treatment of lung cancer.

Fucoidan has been shown to exhibit impressive biological activities, such as antioxidant, immunoregulatory, anti-inflammatory, antitumor, and antithrombotic effects [4]. The degree of sulfation, sulfation pattern, molecular weight (MW), and glycosidic branches of various fucoidans influence the aforementioned biological activities [4]. Sulfate content appears to be the most critical variable [5]. According to a study conducted by Soeda et al. [5], fucoidan derivatives with varying sulfate contents were capable of promoting tissue plasminogen activator (t-PA)-induced plasma clot lysis and preventing the formation of fibrin polymers. These activities were enhanced in direct proportion to the degree of sulfation. In another study by Koyanagi et al. [6], it was shown that oversulfated fucoidans demonstrated greater anti-angiogenic activity compared with native fucoidans, and therefore they were able to inhibit the growth of tumor cells more effectively by suppressing angiogenesis. Moreover, in comparison with native fucoidans, oversulfated fucoidans appeared to show more potent anticancer activity against AGS, a human stomach cancer cell line [7]. The above-mentioned studies indicate that the sulfate content of fucoidans has a significant influence on their biological properties and that the modification of sulfate content could thus potentially enhance said properties. A number of studies have explored the biological activities of oversulfated fucoidans, but relatively little is known about the effects that varying levels of sulfation of fucoidan have on anti-lung cancer activity, and the mechanism involved remains poorly understood.

This investigation is an extension of our previous study, in which a native fucoidan (SC) was created from single-screw extrusion pretreated *Sargassum aquifolium*. Three degraded fucoidan products were developed: SCA (degradation of SC by ascorbic acid), SCH (degradation of SC by hydrogen peroxide), and SCAH (degradation of SC by ascorbic acid + hydrogen peroxide). The results of the study showed that SCA had high cytotoxicity to lung cancer cells as well as a strong ability to suppress Bcl-2 expression in lung cancer cells. Moreover, SCA showed high efficacy with respect to induction of cytochrome *c* release, promotion of late apoptosis of lung cancer cells, and activation of caspase-9 and -3 [8]. In the present study, SCA served as a native fucoidan from which two fucoidan derivatives with different levels of sulfation were generated: SCA-S1 and SCA-S2. Then we analyzed the anticancer activities of SCA, SCA-S1, and SCA-S2 against human lung carcinoma A-549 cells in terms of cell cycle, cytotoxicity, expression of caspase-3, mitochondrial membrane potential (MMP), cytochrome *c* release, Bcl-2 expression, Annexin V/ propidium iodide (PI) staining, and DNA fragmentation, as well as the underlying signaling transduction cascades. To the best of the authors’ knowledge, this is the first study to investigate a potential mechanism of anti-lung cancer activity involving oversulfated fucoidans obtained from single-screw extrusion pretreated *S. aquifolium*. In future research, we intend to explore the clinical applications of oversulfated fucoidans in the treatment and prevention of lung cancer and possibly other cancers.

## 2. Results

### 2.1. Preparation of Oversulfated Fucoidans (SCA-S1 and SCA-S2) and Compositional Analysis

SCA is a degraded fucoidan product, which was previously produced by our laboratory. The results of our in vitro analyses demonstrated that SCA possesses anti-lung cancer properties [8]. We used SCA in the present study as a native fucoidan and created two oversulfated fucoidans termed SCA-S1 and SCA-S2. Table 1 displays the chemical and monosaccharide compositions of SCA, SCA-S1, and SCA-S2. The percentages of sulfate content for SCA, SCA-S1, and SCA-S2 were 13.67 ± 2.19%, 34.67 ± 3.73%, and 60.63 ± 3.69%, respectively. The greater sulfate content in SCA-S1 and SCA-S2 indicates that the addition of sulfate in SCA was successful. The total sugar contents of SCA, SCA-S1, and SCA-S2 ranged from 28.95 ± 0.24% to 41.70 ± 0.91% (*w*/*w*, dry basis). The addition of sulfate to fucoidan generally resulted in a reduction in total sugar content. The fucose contents of SCA, SCA-S1, and SCA-S2 were 35.22 ± 2.79%, 20.36 ± 1.52%, and 12.58 ± 0.46%, respectively. Similarly, the fucose content of fucoidan was found to be lower following oversulfation. These results suggest that the addition of sulfate to fucoidan lowers its total sugar and fucose contents. Table 1 shows the monosaccharide compositions of these fucoidans. The major neutral sugar constituents in SCA were galactose, fucose, and galacturonic acid, while the minor sugar units consisted of xylose, mannose, and glucuronic acid. The monosaccharide composition of fucoidan did not appear to change significantly following oversulfation, although SCA-S1 and SCA-S2 showed a reduction in galacturonic acid. Taken together, the aforementioned results indicate that oversulfated fucoidans had higher sulfate content, lower total sugar and fucose contents, and monosaccharide compositions were altered, albeit only slightly. Our results demonstrated differences in compositions among SCA, SCA-S1, and SCA-S2, and thus further analyses of the biological functions of these fucoidans are warranted.

### 2.2. Structural Analysis of SCA, SCA-S1, and SCA-S2

**Figure 1:**
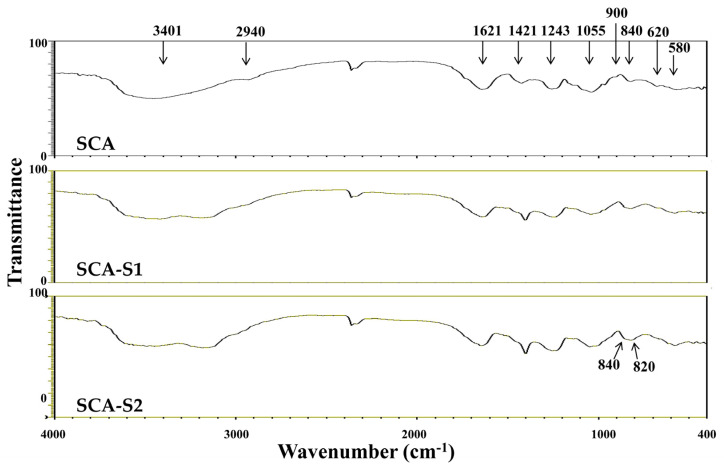
FTIR spectra for SCA, SCA-S1, and SCA-S2. The characteristic peaks at 3401, 2940, 1621, 1421, 1243, 1055, 900, 840, 820, 620, and 580 cm^−1^ are labeled.

**Figure 2:**
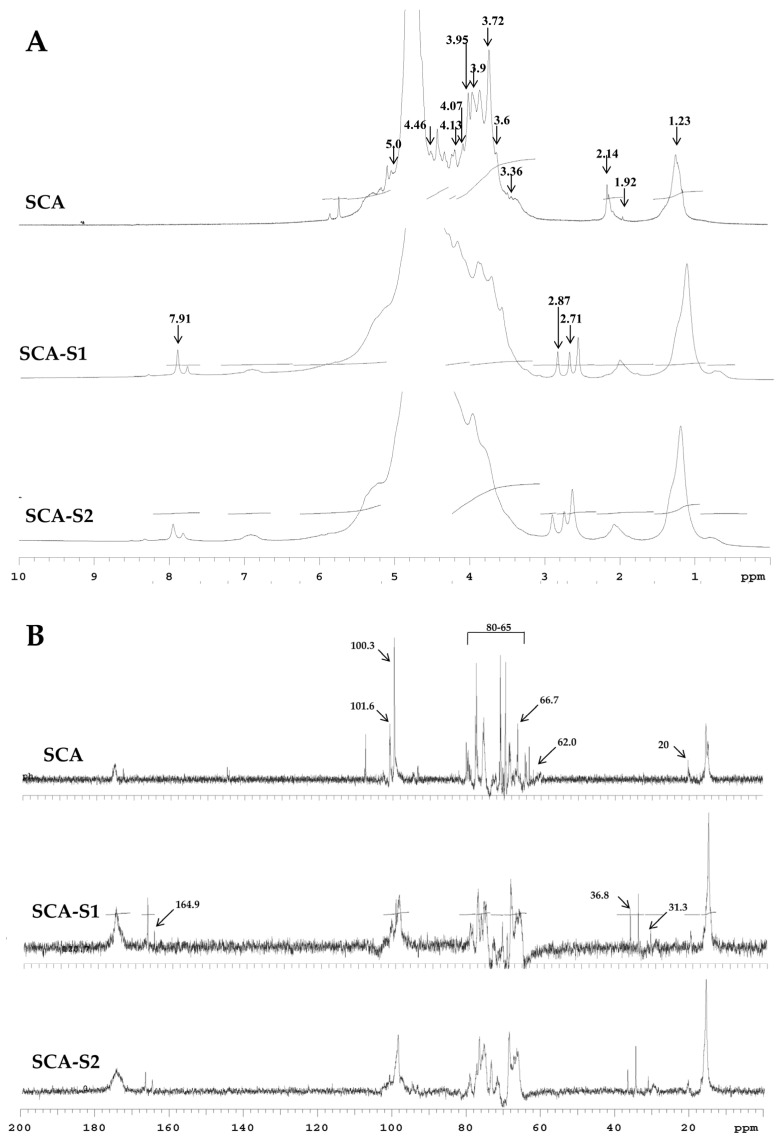
NMR analyses of SCA, SCA-S1, and SCA-S2. (**A**) ^1^H-NMR spectra for SCA, SCA-S1, and SCA-S2. The characteristic peaks at 7.91, 5.0, 4.46, 4.13, 4.07, 3.95, 3.9, 3.72, 3.6, 3.36, 2.87, 2.71, 2.14, 1.92, and 1.23 ppm are indicated. (**B**) ^13^C-NMR spectra for SCA, SCA-S1, and SCA-S2. The characteristic peaks at 164.9, 101.6, 100.3, 80–65, 66.7, 62.0, 36.8, 31.3, and 20 ppm are indicated.

Fourier transform infrared (FTIR) and nuclear magnetic resonance (NMR) techniques were employed to conduct structural analyses of SCA, SCA-S1, and SCA-S2. Figure 1 depicts IR bands at 3401 and 2940 cm^−^^1^ which correspond to the presence of OH and H_2_O stretching vibration and C–H stretching of the pyranoid ring or the C-6 group of fucose and galactose units [9,10]. Absorption bands were detected at 1621 and 1421 cm^−1^ which can be attributed to the scissoring vibration of H_2_O and in-plane ring CCH, COH, and OCH vibrations, characteristic of the absorption pattern of polysaccharide [9,10,11]. The peaks at 1243 and 1055 cm^−^^1^ can be ascribed to the presence of the asymmetric stretching of S=O and the C–O–C stretching vibrations in ring or C–O–H in the glucosidal bond [9,10]. The absorption bands at 900 and 840 cm^−1^ were due to the presence of C1–H bending in the β-anomeric link of galactose and equatorial C–O–S bending vibration of sulfate substituents at the axial C-4 position [12]. The bands at 620 and 580 cm^−1^ may correspond to symmetric and anti-symmetric O=S=O deformations [13]. Figure 2A shows the ^1^H-NMR spectra for SCA, SCA-S1, and SCA-S2. The signals from 5.5 to 5.0 ppm can be attributed to l-fucopyranosyl units [14]. The signal at 4.46 ppm, which was most apparent in SCA, denotes the presence of H-2 in a 2-sulfated fucopyranose residue [14], and the signal at 4.13 ppm (4[H]) indicates the presence of 3-linked α-l-fucose [14]. Signals with a ppm of 4.07/3.95 (6[H]/6′[H]), which were pronounced in SCA, can be explained by the presence of a (1-6)-β-d-linked galacton [15]. Moreover, the signals from 3.9 to 3.6 ppm could be characteristic signals of mannitol [16,17], which is frequently extracted along with fucoidan. The signal obtained at 3.72 ppm may denote the presence of (4[H]) 2,3-linkedα-β-mannose [11], and the signal at 2.14 ppm may indicate methyl protons in *O*-acetyls [11,18], which are frequently detected in algal polysaccharides [18]. The signals at 1.92 (1[H]) and 1.23 ppm (6[H]) demonstrate the existence of alkyl at a sulfonyl-attached proton and an alkane proton in two methyl groups, respectively [19]. Other signals, including 7.91, 2.87, and 2.71 ppm, were detected in SCA-S1 and SCA-S2 and these may correspond to N, N-dimethylformamide (DMF), which is a sulfation reagent utilized in the oversulfate treatment of fucoidan. The ^13^C-NMR spectra (Figure 2B) for SCA, SCA-S1, and SCA-S2 revealed that the prominent signal at 101.6 ppm and peaks between 65–80 ppm correspond to (1-6)-β-d-linked galacton [15]. The signal at 100.3 ppm can be assigned to a (1,3)-linked α-L-fucopyranose residue [17]. The signals at 62.0 and 66.7 ppm signified β-d-galactopyranose residues [20]. Peaks at 19–20 ppm revealed the presence of *O*-acetyl groups [21], which is often visible in algal polysaccharides. Additional signals can be found in SCA-S1 and SCA-S2 including 164.9, 36.8, and 31.3 ppm, which can be assigned to DMF, a sulfation reagent used for oversulfation of fucoidan [22]. In summary, the data pertaining to FTIR, ^1^H NMR, and ^13^C-NMR indicate that SCA, SCA-S1, and SCA-S2 have the characteristic structural features of fucoidan, and that DMF signals could only be detected in SCA-S1 and SCA-S2 using NMR spectra.

### 2.3. SCA, SCA-S1, and SCA-S2 Exhibited Cytotoxic Effects on A-549 Cells

**Figure 3:**
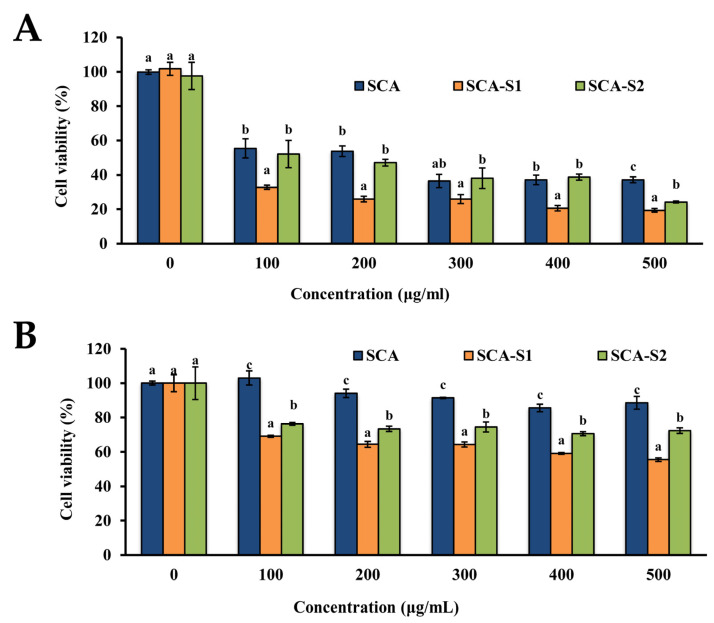
Effects of SCA, SCA-S1, and SCA-S2 on cell viabilities of A-549 and BEAS-2B cells: (**A**) A-549 cells were cotreated with 0–500 µg/mL of SCA, SCA-S1, and SCA-S2 for 48 h, and the cell viability was measured by MTT assays; (**B**) BEAS-2B cells were coincubated with 0–500 µg/mL of SCA, SCA-S1, and SCA-S2 for 48 h, and the cell viability was determined by MTT assays. Experiments were performed in triplicate. Bars with different letters significantly differ at the level of 0.05.

The human lung carcinoma A-549 cell line is considered a useful in vitro model for investigations of the anti-lung cancer effects of fucoidans [8]. Figure 3A shows the cytotoxic effects of SCA, SCA-S1, and SCA-S2 on A-549 cells. All fucoidans, namely SCA, SCA-S1, and SCA-S2, had reduced ratios of live A-549 cells in a dose-dependent manner, and SCA-S1 exhibited more potent cytotoxic effects on A-549 cells compared with those of SCA and SCA-S2. BEAS-2B, a non-cancerous bronchial epithelial cell line, can be used to represent normal human lung cells [23]. Hence, we conducted a similar experiment using BEAS-2B cells to determine whether these fucoidans exert cytotoxic effects on normal cells. As shown in Figure 3B, the results suggest that SCA-S1 conferred the largest cytotoxicities on BEAS-2B cells, followed by SCA-S2 and SCA. This response was similar to that seen in A-549 cells. In addition, SCA, SCA-S1, and SCA-S2 showed lower cytotoxicities to BEAS-2B in comparison with A-549 cells (Figure 3A,B). Moreover, SCA, SCA-S1, and SCA-S2 had survival rates of A-549 cells ranging from 25.9% to 53.8% at a concentration of 200 µg/mL, and of BEAS-2B cells, survival rates ranged from 64.4% to 94.1%, suggesting these fucoidans were less cytotoxic to normal cells. In our preliminary experiment, a treatment time of 48 h was found to be optimal for the induction of cytotoxicity in A-549 cells. As the survival rates of A-549 cells were reduced to less than 50% (approx.) following treatment of these fucoidans, a concentration of 200 µg/mL and a treatment duration of 48 h were adopted for further in vitro anti-lung cancer experiments.

### 2.4. Effects of SCA, SCA-S1, and SCA-S2 on Cell Cycle Profile of A-549 Cells

**Figure 4:**
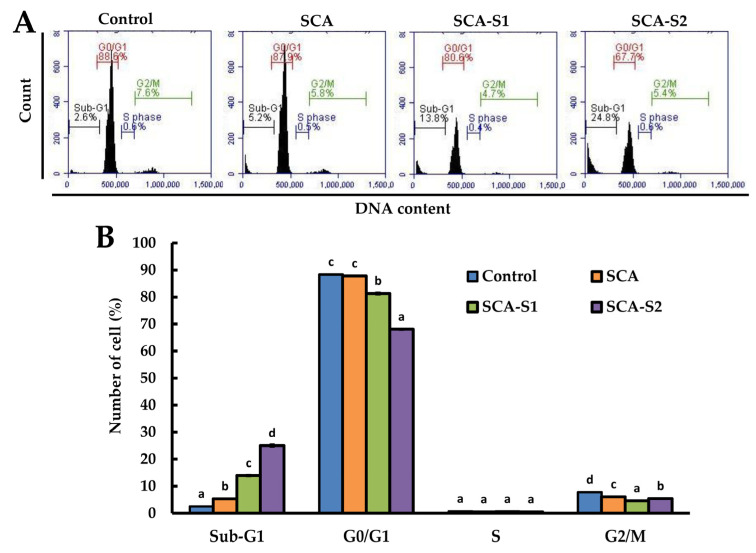
Effects of SCA, SCA-S1, and SCA-S2 treatments on cell cycle profiles of A-549 cells: (**A**) A-549 cells were treated with SCA, SCA-S1, and SCA-S2 at a concentration of 200 µg/mL for 48 h, and cell cycle profiles were measured; (**B**) summary bar graph of three cell cytometric analyses showing the percentages of cells in the sub-G_1_, G_0_/G_1_, S, and G_2_/M phases of the cell cycle according to treatments. Results are shown as mean ± SD of three separate experiments. Differences exist between columns labeled with different letters at the level of 0.05.

Figure 4 shows that when A-549 cells were treated with 200 µg/mL SCA, SCA-S1, and SCA-S2 for 48 h, SCA-S2 had the highest percentage of cells in the sub-*G*_1_ phase (25.0 ± 0.6%), followed by SCA-S1 (13.9 ± 0.4%), SCA (5.33 ± 0.12%), and untreated cells (2.50 ± 0.14%). The cell population in the sub-*G*_1_ phase rose in direct proportion to the induction of DNA fragmentation [24]. As such, SCA-S2 showed the greatest DNA fragmentation (also termed sub-*G*_1_ cell cycle arrest), followed by SCA-S1, SCA, and untreated cells. In summary, all of the tested fucoidans were capable of inducing sub-*G*_1_ cell cycle arrest. SCA-S2 displayed the greatest ability to induce DNA fragmentation of A-549 cells.

### 2.5. Effects of SCA, SCA-S1, and SCA-S2 on Mitochondrial Membrane Potential, Bcl-2 Expression, and Cytochrome c Release of A-549 Cells

**Figure 5:**
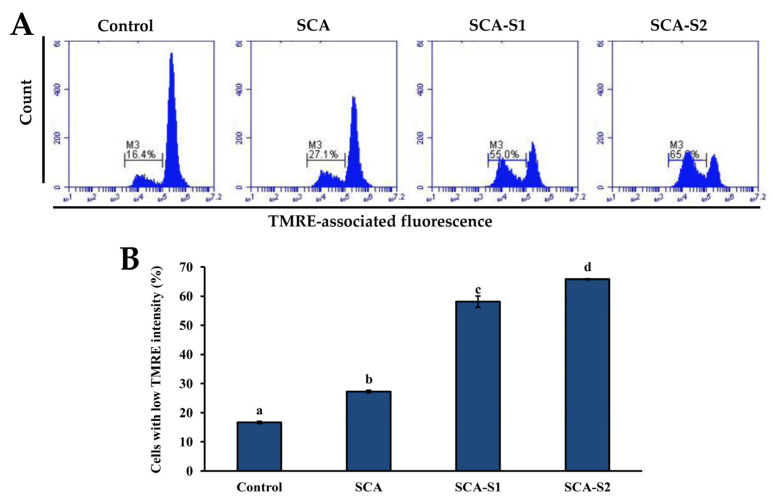
Effects of SCA, SCA-S1, and SCA-S2 treatments on MMP of A-549 cells. A-549 cells were treated with and without 200 µg/mL SCA, SCA-S1, and SCA-S2 for 48 h, and MMP was determined by TMRE staining and flow cytometry. (**A**) Histograms; (**B**) summary bar graph of three cell cytometric analyses showing the percentages of cells with low TMRE intensity according to treatments. Results are shown as mean ± SD of three separate experiments. Differences exist between columns labeled with different letters at the level of 0.05.

**Figure 6:**
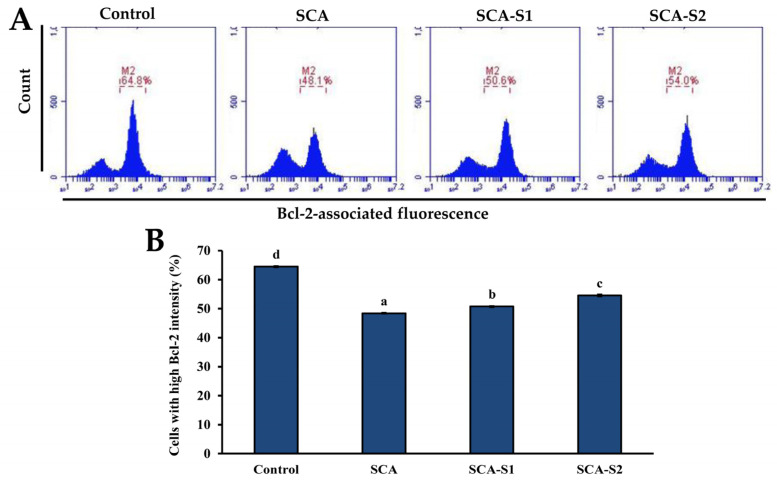
Effects of SCA, SCA-S1, and SCA-S2 treatments on the Bcl-2 expression in A-549 cells. A-549 cells were treated with and without 200 µg/mL SCA, SCA-S1, and SCA-S2 for 48 h, and the level of immunolabeled Bcl-2 was determined by flow cytometry. (**A**) Histograms; (**B**) summary bar graph of three cell cytometric analyses showing the percentages of cells with high Bcl-2 intensity according to treatments. Results are shown as mean ± SD of three separate experiments. Differences exist between columns labeled with different letters at the level of 0.05.

**Figure 7:**
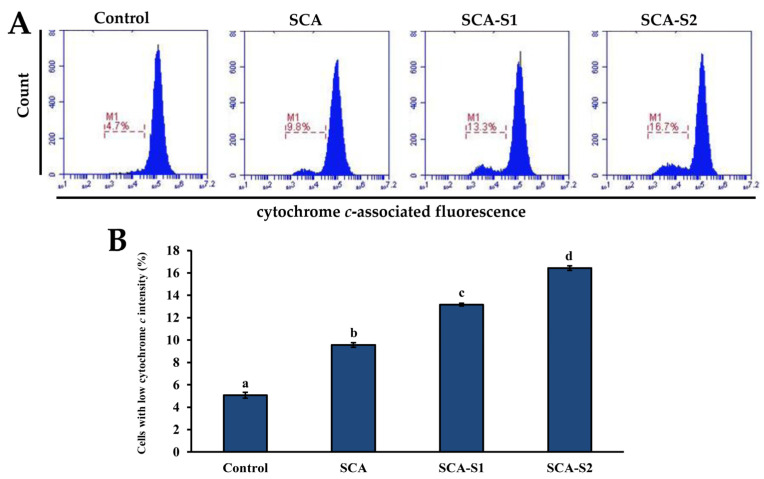
Effects of SCA, SCA-S1, and SCA-S2 treatments on the amount of cytochrome *c* release in A-549 cells. A-549 cells were treated with and without 200 µg/mL SCA, SCA-S1, and SCA-S2 for 48 h, and the level of immunolabeled cytochrome *c* was determined by flow cytometry. (**A**) Histograms; (**B**) summary bar graph of three cell cytometric analyses showing the percentages of cells with low cytochrome *c* intensity according to treatments. Results are shown as mean ± SD of three separate experiments. Differences exist between columns labeled with different letters at the level of 0.05.

It is thought that TMRE binds to active mitochondria owing to its ability to permeate cells in addition to its positive charge. Loss of MMP is directly related to reduced TMRE binding [25]. In Figure 5, the percentage of cells with low TMRE intensity in the control was 16.7 ± 0.4%. Following treatment of A-549 cells with 200 µg/mL SCA, SCA-S1, and SCA-S2 for 48 h, the percentage of cells with low TMRE intensity increased significantly to 27.3 ± 0.4%, 58.2 ± 2.0%, and 65.9 ± 0.2%, respectively (*p* < 0.05), indicating the occurrence of fucoidan-induced mitochondrial dysfunction. Bcl-2 is a member of the anti-apoptotic class of B cell leukemia-2 gene product (Bcl-2) family proteins and it has been postulated that it blocks MMP depolarization [26]. In contrast, suppressed Bcl-2 expression results in cellular apoptosis. In Figure 6 it can be seen that the percentage of cells with high Bcl-2 intensity in the control was 64.5 ± 0.3%. Treatment of A-549 cells with 200 µg/mL SCA, SCA-S1, and SCA-S2 for 48 h resulted in a reduction of the percentage of cells with high Bcl-2 intensity to 48.4 ± 0.2%, 50.7 ± 0.2%, and 54.5 ± 0.4%, respectively, suggesting the occurrence of fucoidan-mediated suppression of Bcl-2. The release of cytochrome *c* from mitochondria is a nearly apoptotic event and is an upstream signal of the mitochondria-dependent apoptotic pathway [27,28]. Figure 7 shows that in the control, the percentage of cells with low cytochrome *c* intensity was 5.07 ± 0.26%. The percentage of cells with low cytochrome *c* intensity significantly increased to 9.57 ± 0.21%, 13.2 ± 0.1%, and 16.4 ± 0.2%, respectively (*p* < 0.05), when A-549 cells were treated with 200 µg/mL SCA, SCA-S1, and SCA-S2 for 48 h, suggesting the involvement of fucoidan-mediated release of cytochrome *c* from mitochondria. In summary, these findings indicate that SCA, SCA-S1, and SCA-S2 induced mitochondria-dependent apoptotic effects, as evidenced by the loss of MMP, release of cytochrome *c*, and suppression of Bcl-2.

### 2.6. Effects of SCA, SCA-S1, and SCA-S2 on Activation of Caspase-3 and DNA Fragmentation of A-549 Cells

**Figure 8:**
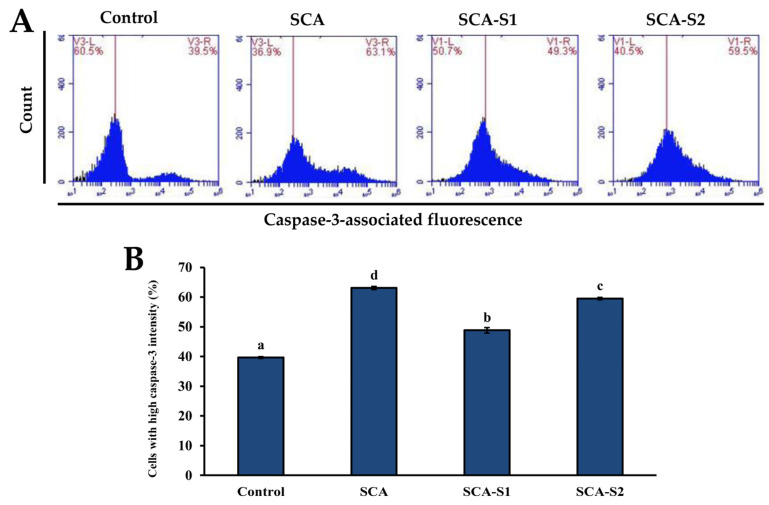
Effects of SCA, SCA-S1, and SCA-S2 treatments on the activation of caspase-3 in A-549 cells. A-549 cells were treated with and without 200 µg/mL SCA, SCA-S1, and SCA-S2 for 48 h, and the level of immunolabeled caspase-3 was determined by flow cytometry. (**A**) Histograms; (**B**) summary bar graph of three cell cytometric analyses showing the percentages of cells with high caspase-3 intensity according to treatments. Results are shown as mean ± SD of three separate experiments. Differences exist between columns labeled with different letters at the level of 0.05.

**Figure 9:**
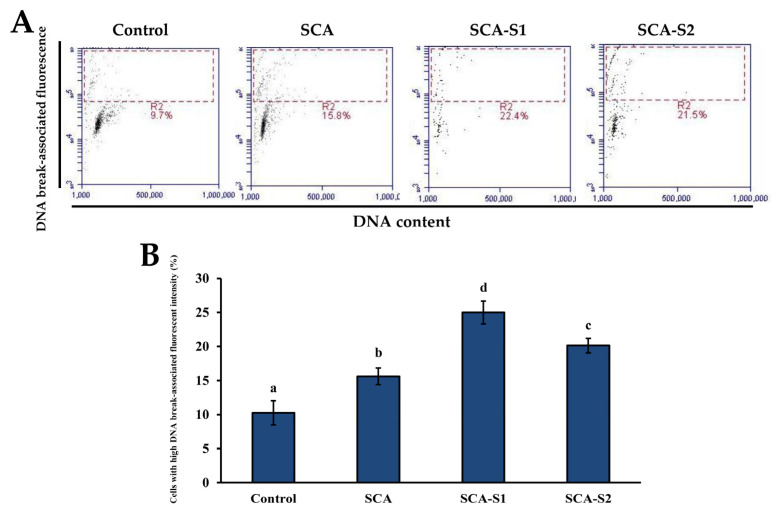
Effects of SCA, SCA-S1, and SCA-S2 treatments on the extent of DNA fragmentation in A-549 cells. A-549 cells were treated with and without 200 µg/mL SCA, SCA-S1, and SCA-S2 for 48 h, and the level of immunolabeled BrdU was determined by flow cytometry. (**A**) Histograms; (**B**) summary bar graph of three cell cytometric analyses showing the percentages of cells with high DNA break-associated fluorescent intensity according to treatments. Results are shown as mean ± SD of three separate experiments. Differences exist between columns labeled with different letters at the level of 0.05.

When cytochrome *c* is released from the mitochondrial intermembrane space, apoptosome formation is triggered, leading to the induction of caspase-9 and caspase-3 activation [29]. In Figure 8, it can be seen that the percentage of cells in the control with high caspase-3 intensity was 39.6 ± 0.3%. Treatment of A-549 cells with 200 µg/mL SCA, SCA-S1, and SCA-S2 for 48 h led to an increase in the percentage of cells with high caspase-3 intensity to 63.1 ± 0.5%, 48.8 ± 0.9%, and 59.5 ± 0.4%, respectively, thus providing evidence that activation of caspase-3 was mediated by fucoidan. Activation of caspase-3 was shown to be a vital component of apoptotic cascades and triggers fragmentation of DNA, resulting in late phase apoptosis [30,31]. Figure 9 demonstrates that the percentage of cells in the control with a high DNA break-associated fluorescent intensity was 10.3 ± 1.8%. Treatment of A-549 cells with 200 µg/mL SCA, SCA-S1, and SCA-S2 for 48 h significantly enhanced the percentage of cells with high DNA break-associated fluorescent intensity by 15.6 ± 1.2%, 25.0 ± 1.7%, and 20.1 ± 1.1% (*p* < 0.05), respectively, suggesting the occurrence of fucoidan-mediated DNA fragmentation.

### 2.7. Effects of SCA, SCA-S1, and SCA-S2 on the Induction of Apoptosis in A-549 Cells

**Figure 10:**
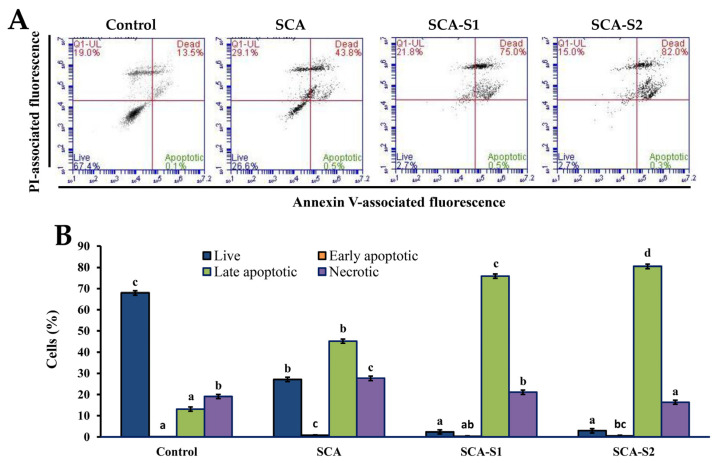
Effects of SCA, SCA-S1, and SCA-S2 treatments on the induction of apoptosis in A-549 cells. A-549 cells were treated with and without 200 µg/mL SCA, SCA-S1, and SCA-S2 for 48 h, and the Annexin V-FITC/PI-stained cells were determined by flow cytometry. (**A**) Histograms; (**B**) summary bar graph of three cell cytometric analyses showing the percentages of Annexin V-FITC/PI-stained cells according to treatments. Results are shown as mean ± SD of three separate experiments. Differences exist between columns labeled with different letters at the level of 0.05.

Loss of plasma membrane asymmetry occurs early in apoptosis, leading to exposure of phosphatidylserine (PS) residues at the outer plasma membrane [32]. The specific binding of Annexin V to PS means that loss of plasma membrane integrity can be used in the detection of apoptosis [32]. The Annexin V-FITC and PI double-staining method is also capable of providing information related to necrotic cells as well as early- and late-stage apoptosis. Figure 10 shows that, in the control, the percentage of live cells was 68.0 ± 0.8%. Furthermore, treatment of A-549 cells with 200 µg/mL SCA, SCA-S1, and SCA-S2 for 48 h led to a decrease in the percentage of live cells to 27.2 ± 1.3%, 2.38 ± 0.83%, and 2.93 ± 0.40%, respectively. Meanwhile, the percentage of late apoptotic cells in the control was 13.2 ± 0.6%. When A-549 cells were subjected to 200 µg/mL SCA, SCA-S1, and SCA-S2 for 48 h, the percentage of late apoptotic cells rose to 45.2 ± 1.0%, 75.9 ± 0.7%, and 80.5 ± 1.1%, respectively. The aforementioned findings clearly demonstrate that A-549 cellular death (primary late apoptosis) was induced by SCA, SCA-S1, and SCA-S2.

### 2.8. SCA, SCA-S1, and SCA-S2 Induced Dephosphorylation of Akt, mTOR, and S6 in A-549 Cells

**Figure 11:**
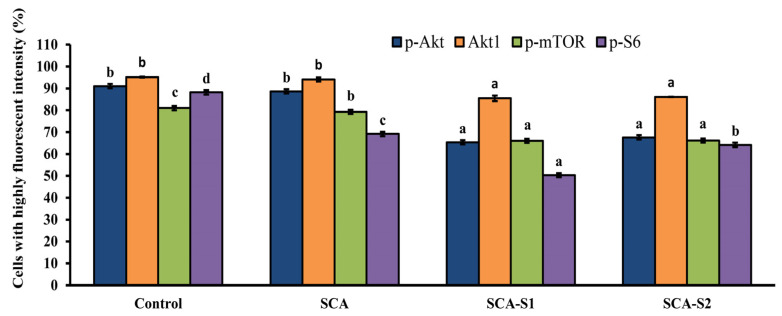
Effects of SCA, SCA-S1, and SCA-S2 treatments on the levels of p-Akt, Akt1, p-mTOR, and p-S6 in A-549 cells. A-549 cells were treated with and without 200 µg/mL SCA, SCA-S1, and SCA-S2 for 48 h, and the cells with highly fluorescent intensity were determined by flow cytometry. The bar graph summary of three cell cytometric analyses shows the percentages of cells with highly fluorescent intensity according to treatments. Results are shown as mean ± SD of three separate experiments. Differences exist between columns labeled with different letters at the level of 0.05.

Figure 11 depicts the percentage of cells in the control with high p-Akt intensity: 91.0 ± 0.2%. When A-549 cells were exposed to 200 µg/mL SCA, SCA-S1, and SCA-S2 for 48 h, the percentage of cells with high p-Akt intensity fell to 88.6 ± 1.9%, 65.3 ± 3.0%, and 67.6 ± 3.5%, respectively. Moreover, the percentage of cells with high Akt1 intensity in the control was 95.2 ± 0.3%. When A-549 cells were subjected to 200 µg/mL SCA, SCA-S1, and SCA-S2 for 48 h, the percentage of cells with high Akt1 intensity was 94.1 ± 1.0%, 85.4 ± 1.2%, and 86.7 ± 0.1%, respectively. The percentage of cells with high p-mTOR intensity in the control was 81.0 ± 0.4%. Following the treatment of A-549 cells with 200 µg/mL SCA, SCA-S1, and SCA-S2 for 48 h, the percentage of cells with high p-mTOR intensity was reduced to 79.3 ± 0.4%, 66.0 ± 0.8%, and 66.1 ± 0.8%, respectively. In the control, the percentage of cells with high p-S6 intensity was 88.2 ± 0.7%. The percentage of cells with high p-S6 intensity dropped to 69.2 ± 0.9%, 50.3 ± 2.3%, and 64.1 ± 1.2%, respectively, when A-549 cells had been treated with 200 µg/mL SCA, SCA-S1, and SCA-S2 for 48 h. The data above provide clear evidence of fucoidan-mediated dephosphorylation of Akt, mTOR, and S6 in A-549 cells.

## 3. Discussion

A number of studies in the literature have indicated that oversulfated fucoidans exhibit greater anti-angiogenic activity compared to native fucoidans, and therefore by mitigating angiogenesis they can inhibit the growth of tumor cell growth more efficiently [6]. The wide range of anticancer activity seen among the various oversulfated fucoidan derivatives can probably be explained by differences in sulfate content [7]. The present study is an extension of our previous work on a native fucoidan (SC, extracted from *Sargassum aquifolium*) and three fucoidan hydrolysates, which we developed, termed SCA (degradation of SC by ascorbic acid), SCH (degradation of SC by hydrogen peroxide), and SCAH (degradation of SC by ascorbic acid + hydrogen peroxide). Our analyses determined that SCA was a suitable candidate for further development as an adjuvant therapy for lung cancer [8]. In the current study, we oversulfated SCA and developed two oversulfated fucoidans termed SCA-S1 and SCA-S2. The sulfate contents of SCA, SCA-S1, and SCA-S2 were 13.67 ± 2.19%, 34.67 ± 3.73%, and 60.63 ± 3.69%, respectively (Table 1). In a study by Cho et al. [7], the addition of sulfate groups increased the sulfate content of the low molecular weight fucoidan (F_5–30K_ fraction) up to 56.8%. The sulfate content of oversulfated F_5–30K_ fraction was similar to that of SCA-S2, indicating that oversulfation of fucoidan is capable of yielding a sulfate content reaching as high as 60% (approx.). A summary of the chemical properties of SCA, SCA-S1, and SCA-S2 is presented in Table 1. Increases in sulfate content may result in a proportional decrease in the fucose content of fucoidan, suggesting that oversulfation may modify the fundamental structure of fucoidan. The FTIR spectra for SCA, SCA-S1, and SCA-S2 (Figure 1) depict broader peak areas at 1243 (the asymmetric stretching of S=O), 840 (C–O–S bending vibration of sulfate substituents at the axial C-4 position), and 620/580 (the symmetric and anti-symmetric O=S=O deformations) cm^−1^ in SCA-S1 and SCA-S2, which are indicative of higher sulfate contents in SCA-S1 and SCA-S2. Moreover, Partankar et al. [33] reported that the sulfate peaks at around 820 and 840 cm^−1^ correspond to the equatorial C-2 and axial C-4 positions, respectively. In Figure 1, SCA showed a strong peak at around 840 cm^−1^, which indicates that the sulfates had largely been substituted at the C-4 position. In contrast, the oversulfated SCA-S1 and SCA-S2 both showed a pronounced peak at 840 cm^−1^ with a shoulder at 820 cm^−1^ (Figure 1), providing evidence of a 2,4 disubstitution of the sulfate groups. These findings are in line with previously reported studies [7], showing that sulfation resulted in the emergence of a shoulder at 820 cm^−1^ alongside the main peak at 840 cm^−1^ in the IR spectra, indicative of 2,4 disulfation. NMR spectra can be used to further evaluate the structural characteristics of oversulfated fucoidans. After oversulfation, the ^1^H-NMR and ^13^C-NMR spectra of SCA, SCA-S1, and SCA-S2 (Figure 2) were found to be different. Nonetheless, the characteristic peaks of fucoidan were also detected in SCA, SCA-S1, and SCA-S2. Sulfur trioxide *N*,*N*-dimethylformamide complex (SO_3_-DMF) (sulfating agents) in formamide (FA) solution was used to sulfate SCA-S1 and SCA-S2. Of note, the specific ^1^H-NMR and ^13^C-NMR signals representing DMF were detected in SCA-S1 and SCA-S2 (Figure 2), demonstrating the presence of sulfate groups in the fucoidan backbones.

In a previous study, we showed that the native fucoidan (SC) and fucoidan hydrolysates (SCA, SCH, and SCAH) lowered the ratios of live A-549 cells, and SCA, SCH, and SCAH conferred stronger cytotoxic effects on A-549 cells compared with SC [8]. However, all of the tested fucoidans (SC, SCA, SCH, and SCAH) showed less potent cytotoxic effects against normal BEAS-2B cells compared with that of A-549 cells [8]. Although there are a number of available human lung cancer cell lines that can be used to establish a tumor model, such as A-549, H-460, H-1299, H-1650, H-358, and HCC-827 [34], A-549 cells are adenocarcinomic human alveolar basal epithelial cells, which are widely used in models for the study of lung cancer and the development of drug therapies against it [8,35]. Moreover, A-549 cells are easy to maintain and grow faster compared with other lung cancer cells. Thus, A-549 cells were utilized in the present study to evaluate the anticancer effect of fucoidan extracts. In the current study, the most potent cytotoxic effects on A-549 cells were observed in SCA-S1 among the tested fucoidans (Figure 3A). The analyses of the cytotoxicities of SCA-S1 to A-549 cells and BEAS-2B cells at the concentration of 500 µg/mL revealed a survival rate of 19.3 ± 1.0% in A-549 cells, but in BEAS-2B cells the survival rate was 55.5 ± 1.0%, showing that SCA-S1 had a toxic effect on normal cells that was 2.9-fold (55.5/19.3 = 2.9) lower (Figure 3). Likewise, SCA-S2 was shown to have an approximately 3.0-fold (72.4/24.2 = 3.0) lower toxic effect on normal cells (Figure 3). In a study by Cho et al. [7], it was found that fucoidan (F_>30K_ fraction, sulfate content = 41.2%) exhibited 50% anticancer activity against AGS, a human stomach cancer cell line, at a concentration exceeding 800 µg/mL. SCA-S1 possessed a sulfate content of 34.7%, which was similar to that in the F_>30K_ fraction, but it showed a 50% anticancer activity against A-549 cells at a concentration of 74.4 µg/mL. While the cancer cell lines examined in the two aforementioned studies were different, the results still show that SCA-S1 confers a strongly potent effect against cancer cells.

Cell cycle analysis can be employed to assess the growth inhibitory effects of SCA, SCA-S1, and SCA-S2 on A-549 cells. Flow cytometry is a rapid technique that can be used to identify compounds capable of selective or preferential eradication of cancer cells by altering regulation of the cell cycle and/or inducing apoptosis. Treatment of cells with an apoptosis-inducing agent can lead to DNA fragmentation, which can also be analyzed by flow cytometry [24]. Small fragments of DNA can be eluted by washing with PBS. Any cells that have lost DNA will not be stained as obviously using PI stain and will appear to the left of the *G*_1_ peak (the so-called sub-*G*_1_ peak). Figure 4 shows that SCA-S2 had the highest percentage of cells in the sub-*G*_1_ phase, followed by SCA-S1 and SCA. In Table 1, SCA-S2 had the greatest amount of sulfate, followed by SCA-S1 and SCA. Hence, increases in the sulfate content of fucoidan may promote the induction of sub-*G*_1_ cell cycle arrest in a proportional manner.

MMP plays a vital role in cellular energy production (ATP) and in maintaining homeostasis within the cell [36]. MMP disruption is indicative of mitochondria dysfunction in the transduction of an apoptotic signal [37]. In Figure 5, SCA-S2 exerted the strongest effect in terms of induction of mitochondrial dysfunction in A-549 cells, followed by SCA-S1 and SCA. This trend was correlated with sulfate contents (Table 1). Bcl-2, an anti-apoptotic protein, has been postulated to block MMP depolarization, which in turn mitigates the activation of downstream apoptotic molecules, such as cytochrome *c*, AIF, and Smac/Diablo [26]. Moreover, suppression of Bcl-2 expression leads to cellular apoptosis. Figure 6 shows that all of the fucoidans were capable of suppressing the expression of Bcl-2, compared with the control. Release of cytochrome *c* from the mitochondria is a nearly apoptotic event in the mitochondria-dependent apoptotic pathway [38,39]. According to Figure 7, SCA-S2 showed the greatest cytochrome *c* release in A-549 cells, followed by SCA-S1 and SCA. This trend was in proportion to the sulfate contents of the fucoidans (Table 1). Taken together, these results indicate that induction of apoptosis by SCA, SCA-S1, and SCA-S2 was largely via a mitochondria-dependent apoptotic pathway. The sulfate content of fucoidan appears to play a key role in apoptotic cell death. The intrinsic pathway (mitochondria pathway) comprises a serial process involving loss of MMP, release of cytochrome *c* into the cytoplasm, formation of an apoptosome complex, culminating in the activation of caspase-3 [40,41]. Furthermore, the activation of caspase-3 plays a pivotal role in DNA fragmentation, which occurs in late phase apoptosis [31]. All of the fucoidans promoted the activation of caspase-3, as compared to the control, as shown in Figure 8. In Figure 9, the results show that the degree of DNA fragmentation in A-549 cells was enhanced following treatment with SCA, SCA-S1, and SCA-S2. In short, the oversulfated fucoidans exhibited greater DNA fragmentation compared with SCA and the control. Moreover, Annexin V-FITC/PI double staining revealed these fucoidans were likely responsible for A-549 cell death (largely involving late apoptosis), and compared with SCA and the control, oversulfated fucoidans had greater numbers of late apoptotic cells (Figure 10).

Activation of the Akt/mTOR/S6 signaling is common in a wide range of cancers [42,43]. It has been shown that commercialized fucoidan extract from *Fucus vesiculosus* suppressed p-Akt and p-mTOR in A-549 cells in a dose- and time-dependent manner [44]. In the current investigation, we found that SCA, SCA-S1, and SCA-S2 suppressed levels of p-Akt, p-mTOR, and p-S6 in comparison with the untreated control. Moreover, the results indicated that SCA-S1 and SCA-S2 showed greater effectiveness with respect to reducing expressions of p-Akt, p-mTOR, and p-S6 compared with SCA. These results provide clear evidence that the oversulfated fucoidan enhances the anticancer activity (particularly against lung cancer) and the underlying mechanism involves the Akt/mTOR/S6 pathway. These encouraging findings could be useful in the future development of fucoidans with extensive sulfate substitution with a view to boosting their anticancer properties. While the precise mechanism has yet to be fully elucidated, it is reasonable to postulate that the elevated negative charge induced by oversulfation enhances the interaction with particular proteins, including plasmatic proteins, adhesion proteins, and growth factors that play a role in cell proliferation, thereby facilitating cell growth suppression [7,45]. Further research is required to gain a complete understanding of the underlying mechanism of action of oversulfated fucoidans using other lung cancer cell lines, and to conduct in vivo models to investigate the upstream and downstream targeting molecules of signaling pathways in lung cancer.

## 4. Materials and Methods

### 4.1. Materials

Samples of *Sargassum aquifolium* were collected from Kenting (Pingtung, Taiwan). After washing and drying, samples were sealed in aluminum foil bags and kept at 4 °C until use. l-fucose, d-galactose, d-glucuronic acid, d-galacturonic acid, d-xylose, d-mannose, dimethyl sulfoxide (DMSO), potassium bromide (KBr), 2,2,2-Trifluoroacetic acid (TFA), and 3-(4,5-dimethylthiazol-2-yl)-2,5-diphenyltetrazolium bromide (MTT) were obtained from Sigma-Aldrich (St. Louis, MO, USA). Ham’s F12K medium, DMEM medium, trypsin/EDTA, fetal bovine serum (FBS), penicillin, and streptomycin were purchased from Gibco Laboratories (Grand Island, NY, USA). TMRE was obtained from Molecular Probes, Invitrogen Corp. (Carlsbad, CA, USA). Unless otherwise stated, other reagents were purchased from Sigma-Aldrich (St. Louis, MO, USA).

### 4.2. Sulfation of Fucoidan

SCA was produced in accordance with the methods described in our previous studies [8]. Sulfation of SCA was performed according to the method described by Wang et al. [22]. The sulfation reagent, SO_3_-DMF, was obtained by dropping 20 mL of chlorosulfonic acid into 100 mL of *N*,*N*-dimethylformamide under cooling in an ice-water bath. Dry SCA (0.1 g) was added to 10 mL formamide (FA), and the mixture was stirred at RT for 30 min in order to disperse it into the solvent. Then 10 mL SO_3_-DMF reagent (for SCA-S1) or 20 mL SO_3_-DMF reagent (for SCA-S2) was added. After reaction at RT for 4 h, the solution was neutralized to pH = 7.0 with 1 mol/L NaOH solution and dialyzed against distilled water for 24 h using 1000 Da MW cutoff dialysis membranes. The remnant was concentrated and lyophilized to obtain SCA-S1 and SCA-S2.

### 4.3. Analytical Methods

The fucose content was estimated using the protocol described by Huang et al. [46] and l-fucose was used as the standard. For the determination of the sulfate content, the sample was firstly hydrolyzed with 1 N HCl solution for 5 h at 105 °C. The hydrolysate was quantified to determine the percentage of sulfate composition using Dionex ICS-1500 Ion Chromatography with IonPac AS9-HC column at a flow rate of 1 mL/min at 30 °C with conductometric detection. The eluent was 9 mM Na_2_CO_3_, and K_2_SO_4_ was utilized as standard. Total sugar content was assayed using a phenol-sulfuric acid method using l-fucose as the standard.

### 4.4. Monosaccharide Composition Analysis

For the determination of monosaccharide composition, the sample was first hydrolyzed with 2 M trifluoroacetic acid (TFA) for 4 h at 110 °C. After removing the residual acid, the standard sugars and sample were pre-column derivatized with 1-phenyl-3-methyl-5-pyrazolone (PMP) for 100 min at 70 °C. The resulting solutions were extracted with chloroform three times. Then the PMP derivatives were eluted with a mixture of 0.1 M phosphate buffer (pH 6.7) and acetonitrile in a ratio of 83:17 (*v*/*v*, %) at a flow rate of 1 mL/min on a reversed-phase Inspire™ C18 (250 × 4.6 mm, 5 µm) column with detection at 245 nm. l-fucose, d-galactose, d-glucuronic acid, d-galacturonic acid, d-xylose, and d-mannose were used as standards.

### 4.5. FTIR Spectroscopy

The FTIR spectra were analyzed according to a protocol described in Huang et al. [47]. In brief, the sample was ground evenly with KBr (1:50, *w*/*w*, %) until particles measured less than 2.5 µm in size. The transparent KBr pellets were prepared at 500 kg/cm^2^ under vacuum conditions. The FTIR spectra were obtained using an FT-730 spectrometer (Horiba, Kyoto, Japan). The signals were automatically collected using 60 scans over the range of 4000–400 cm^−1^ at a resolution of 16 cm^−1^ and were compared to a background spectrum collected from the KBr alone.

### 4.6. NMR Spectroscopy

The fucoidan sample was dissolved with 99.9% D_2_O in an NMR tube and the NMR spectra were recorded using a Varian VNMRS-700 NMR spectrometer (Varian, Lexington, MA, USA).

### 4.7. Cell Culture

A-549 (human lung carcinoma, BCRC 60074, cultured in complete Ham’s F12K medium) and BEAS-2B (human bronchial epithelial cells, ATCC CRL-9609, cultured in complete DMEM medium) were obtained from the BCRC (Bioresource Collection and Research Center, Hsinchu, Taiwan) and the ATCC (American Type Culture Collection, Manassas, VA, USA), respectively. All cells were cultured in a 37 °C humidified 5% CO_2_ atmosphere, and the medium was changed every two to three days.

### 4.8. Evaluation of Cytotoxic Activity

The cytotoxic activity of the fucoidan derivatives was measured using the MTT assay. Cells were cultured in medium at 37 °C in a humidified atmosphere with 5% CO_2_ for 24 h. The stock solution of fucoidan extract was prepared by dissolving it in phosphate-buffered saline (PBS) to a concentration of 20 mg/mL. The medium was then removed and the cells were treated with different concentrations of fucoidan extracts by diluting the stock solution with serum-free medium. After 48 h treatment, cells were washed with PBS once, and MTT reagent (0.1 mg/mL) was added. After a 4 h incubation, isopropanol was added and thoroughly mixed by pipetting to dissolve the formazan. The resultant solution was measured by absorption at 560 nm using a spectrophotometer. The cell viability was expressed as a percentage of MTT reduction.

### 4.9. Flow Cytometry-Based Analyses

In all flow cytometry-based analyses, cells (4 × 10^4^ cells/mL) were incubated without (as a non-treated control) and with 200 µg/mL tested samples for 48 h, and then cells were trypsinized and rinsed with PBS to obtain cell samples. Then, each flow cytometry-based analysis was performed according to the following procedures.

The cell cycle analysis was performed according to the method described previously [48]. Briefly, A-549 cells were collected, washed twice with PBS, resuspended in 70% (*v*/*v*) ethanol, and stored at 4 °C for at least 2 h. The cells were then washed with staining buffer twice, and stained with 25 µg/mL RNase A. After staining with RNase A for 15 min, the cells were stained with 50 µg/mL PI solution, and flow cytometry-based analysis was performed.

For the MMP analysis, the assay was performed using the method of Yang et al. [49]. Briefly, single cell suspensions were washed twice with PBS and incubated, in the dark, for 20 min at 37 °C with TMRE (100 nM). After labeling, cells were washed and resuspended for flow-cytometric measurement in staining solution.

The Bcl-2 assay was done according to the method described by Yang et al. [49]. In brief, single-cell suspensions were fixed using fixation buffer at 37 °C for 20 min. The cells were subsequently permeabilized using permeabilization buffer, and incubated, in the dark, for 1 h at RT with FITC (fluorescein isothiocyanate)-labeled anti-Bcl-2 antibody (1:25, *v*/*v*). After labeling, cells were washed and resuspended for flow-cytometric measurement in staining solution.

The analysis of cytochrome *c* release was conducted according to the protocol described by Huang et al. [12]. Briefly, single-cell suspensions were fixed using fixation buffer at 37 °C for 20 min. The cells were subsequently permeabilized using permeabilization buffer and incubated in the dark for 1 h at RT with FITC-labeled anti-cytochrome *c* antibody (1:10, *v*/*v*). After labeling, cells were washed and resuspended for flow-cytometric measurement in staining solution.

For activated caspase-3 analysis, the method of Huang et al. [12] was employed. Briefly, single-cell suspensions were incubated, in the dark, for 1 h at 37 °C with FITC-DEVD-FMK solution. After labeling, cells were washed and resuspended for flow-cytometric measurement in staining solution.

For DNA fragmentation analysis, the procedure was conducted using the method described by Shiao et al. [50]. Briefly, A-549 cells were harvested and fixed with 4% paraformaldehyde, washed, and then incubated with 70% ice-cold ethanol at −20 °C overnight. Cells were washed with wash buffer, followed by the addition of BrdU, and then incubated with FITC-conjugated anti-BrdU antibody at RT for 30 min in the dark. After staining, the cells were resuspended in staining buffer for flow analysis.

The Annexin V-FITC/PI staining analysis was performed with an Annexin V-FITC apoptosis detection kit following the protocol described by Yang et al. [49]. Briefly, single-cell suspensions were incubated for 15 min at RT in the dark with Annexin V-FITC (1:20, *v*/*v*) and PI (1:20, *v*/*v*). After labeling, cells were washed and resuspended for flow-cytometric measurement in staining solution.

For phosphorylated Akt, mTOR, and S6 analyses, as well as Akt1, were done using the techniques described by Huang et al. [51]. In brief, single-cell suspensions were fixed using fixation buffer at 37 °C for 1 h. The cells were then incubated at RT for 1 h in the dark with APC (allophycocyanin)-conjugated anti-Akt1 antibody (1:50, *v*/*v*), FITC-conjugated anti-phospho-Akt (Ser473) antibody (1:20, *v*/*v*), PE (phycoerythrin)-conjugated anti-phospho-mTOR (Ser2448) antibody (1:20, *v*/*v*), or PE-conjugated anti-phospho-S6 (Ser235, Ser236) antibody (1:20, *v*/*v*). After labeling, cells were washed and resuspended for flow-cytometric measurement in staining solution. All of the abovementioned flow cytometric analyses were performed with a BD Accuri C6 flow cytometer (San Jose, CA, USA). All of the flow data were analyzed using BD Accuri C6 software.

### 4.10. Statistical Analysis

All data are expressed as mean ± SD (*n* = 3). Comparisons between different groups were performed by ANOVA followed by Duncan’s multiple range test. A *p*-value less than 0.05 was considered statistically significant.

## 5. Conclusions

In this investigation, we successfully produced three fucoidans (SCA, SCA-S1, and SCA-S2) from *Sargassum aquifolium*, which contained different levels of sulfate content. Comparisons of SCA, SCA-S1, and SCA-S2 revealed differences in chemical compositions and structural features as a result of oversulfation treatment. The mitochondrion-dependent pathway was predominant in SCA-, SCA-S1-, and SCA-S2-induced apoptosis of A-549 cells as evidenced by the analyses of mitochondrial membrane potential (MMP), cytochrome *c* release, Bcl-2 expression, activation of caspase-3, and DNA fragmentation. Moreover, we demonstrated that oversulfation of fucoidan enhanced its activity against lung cancer cells and determined that the underlying mechanism likely involves the Akt/mTOR/S6 pathway. Our results indicate that oversulfated fucoidans hold considerable promise for further development for application as an adjuvant therapy in the treatment of cancer, especially lung cancer.

## Figures and Tables

**Table 1:** Compositional analysis of SCA, SCA-S1, and SCA-S2.

**Chemical Composition**	**SCA ^2^**	**SCA-S1 ^2^**	**SCA-S2 ^2^**
Sulfate (%) ^1^	13.67 ± 2.19 ^a^	34.67 ± 3.73 ^b^	60.63 ± 3.69 ^c^
Total sugar (%) ^1^	41.70 ± 0.91 ^c^	28.95 ± 0.24 ^a^	35.08 ± 0.21 ^b^
Fucose (%) ^1^	35.22 ± 2.79 ^c^	20.36 ± 1.52 ^b^	12.58 ± 0.46 ^a^
**Monosaccharide Composition (Molar Ratio)**	**SCA**	**SCA-S1**	**SCA-S2**
Fucose	1	1	1
Galactose	0.30	0.28	0.27
Glucuronic acid	0.01	ND ^3^	ND
Galacturonic acid	0.11	ND	ND
Mannose	0.05	0.05	0.05
Xylose	0.05	ND	0.04

^1^ Total sugars (%), fucose (%), and sulfate (%) = (*g*/*g*, dry basis) × 100; ^2^ Experiments were performed in triplicate; values in the same row with varying letters differ (*p* < 0.05); ^3^ ND: not detected.

## Data Availability

Data is contained within the article.
